# Successful treatment of refractory cancer therapy-induced thrombocytopenia in a breast cancer patient using romiplostim N01: A case report

**DOI:** 10.1097/MD.0000000000043665

**Published:** 2025-08-08

**Authors:** Peng Liu, Enlai Dong, Yiming Jiang, Lin Chen, Bo Xie, Jiajun Ling

**Affiliations:** a Department of Oncology, General Hospital of Southern Theater Command, Guangzhou, Guangdong Province, China; b Department of Pathology, General Hospital of Southern Theater Command, Guangzhou, Guangdong Province, China; c School of Chinese Materia Medica, Guangzhou University of Chinese Medicine, Guangzhou, China.

**Keywords:** breast cancer, chemotherapy-induced thrombocytopenia, cytopenia, romiplostim N01, thrombopoietin receptor agonist

## Abstract

**Rationale:**

Cancer therapy-induced thrombocytopenia (CTIT) is a frequent complication in cancer treatment, with severe cases posing risks of bleeding and hindering therapeutic progress.

**Patient concerns:**

A breast cancer patient developed refractory CTIT following multiple lines of chemotherapy. Despite treatment with various thrombopoietic agents, including recombinant human thrombopoietin, eltrombopag, avatrombopag, steroids, and platelet transfusions, platelet counts remained below 50 × 10^9^/L, with a nadir of 6 × 10^9^/L.

**Diagnoses:**

Bone marrow biopsies revealed an absence of megakaryocytes, supporting the diagnosis of CTIT.

**Interventions:**

The patient received 2 doses of romiplostim N01.

**Outcomes:**

The romiplostim N01 resulted in a rapid and sustained normalization of platelet counts. Platelet levels were maintained between 251 × 10^9^/L and 304 × 10^9^/L during follow-up, allowing uninterrupted anticancer therapy and achieving stable disease for the primary tumor.

**Lessons:**

Romiplostim N01 effectively managed refractory CTIT in this case, showing rapid and sustained platelet recovery. This case demonstrates the potential of romiplostim N01 as a potential effective treatment for refractory CTIT.

## 1. Introduction

Chemotherapy-induced thrombocytopenia (CIT) presents a significant clinical challenge, as declining platelet counts can lead to increased bleeding risks^[1,2]^ and interruptions in cancer treatment.^[3]^ CIT has recently evolved into the broader concept of cancer therapy-induced thrombocytopenia (CTIT), which encompasses thrombocytopenia resulting from various cancer treatments, including chemotherapy, radiotherapy, targeted therapy, and immunotherapy. While conventional interventions, such as recombinant human thrombopoietin (rhTPO) and platelet transfusions, are often employed,^[4]^ their efficacy is limited in cases of refractory CTIT, where platelet counts remain critically low despite various treatments. This refractory state not only jeopardizes patient safety but also undermines the timely delivery of cancer therapies, adversely impacting outcomes. Here, we report a case of refractory CTIT in which traditional treatments were ineffective. The administration of romiplostim N01, a thrombopoietin receptor agonist (TPO-RA), resulted in a rapid and sustained normalization of platelet counts, enabling uninterrupted cancer therapy.

## 2. Case report

**Figure 1.**
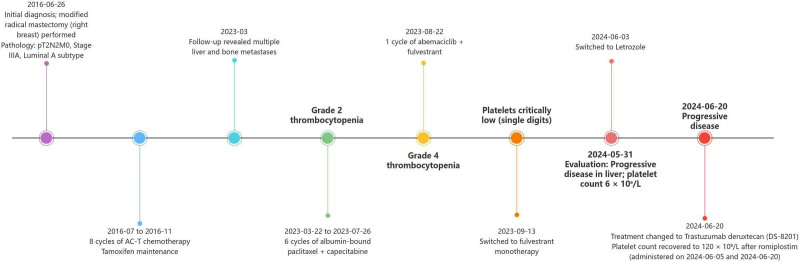
Timeline of clinical course detailing key treatment phases and therapeutic interventions.

**Figure 2.**
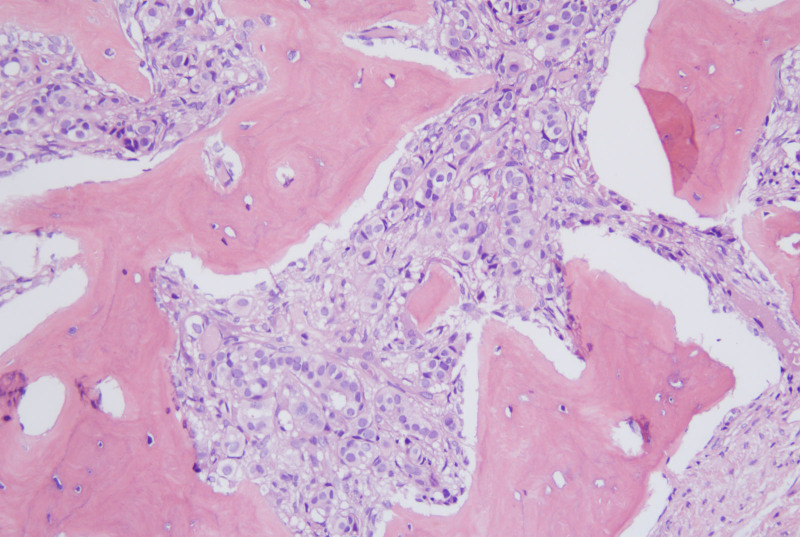
Bone marrow biopsy from the right iliac crest (September 2023) showing absence of megakaryocytes and presence of atypical glandular hyperplasia, consistent with metastatic moderately differentiated adenocarcinoma (magnification ×200).

A 45-year-old woman with a history of right breast invasive ductal carcinoma (grade 2), diagnosed in 2016, underwent a modified radical mastectomy. Postoperative pathology confirmed Luminal A subtype breast cancer (pT2N2M0, stage IIIA), for which she received adjuvant treatment comprising 6 cycles of AC-T chemotherapy (doxorubicin and cyclophosphamide followed by paclitaxel) and tamoxifen maintenance therapy. In March 2023, disease relapse was noted with liver and bone metastases, confirmed on biopsy as Luminal A subtype metastatic breast cancer (rT0N0M1, stage IV). The patient underwent 6 cycles of docetaxel and capecitabine, achieving a partial response. During this regimen, she developed grade 2 CTIT, which initially responded to supportive therapy (Fig. 1). However, following completion of chemotherapy and transition to endocrine maintenance therapy, CTIT worsened to grade 3 to 4, with platelet counts persistently below 50 × 10^9^/L despite dose reductions and successive use of rhTPO, eltrombopag, avatrombopag, steroids, and multiple platelet transfusions. A bone marrow biopsy in September 2023 revealed the absence of megakaryocytes and the presence of atypical cells with glandular arrangements, consistent with metastatic moderately differentiated adenocarcinoma (Fig. 2).

**Figure 3.**
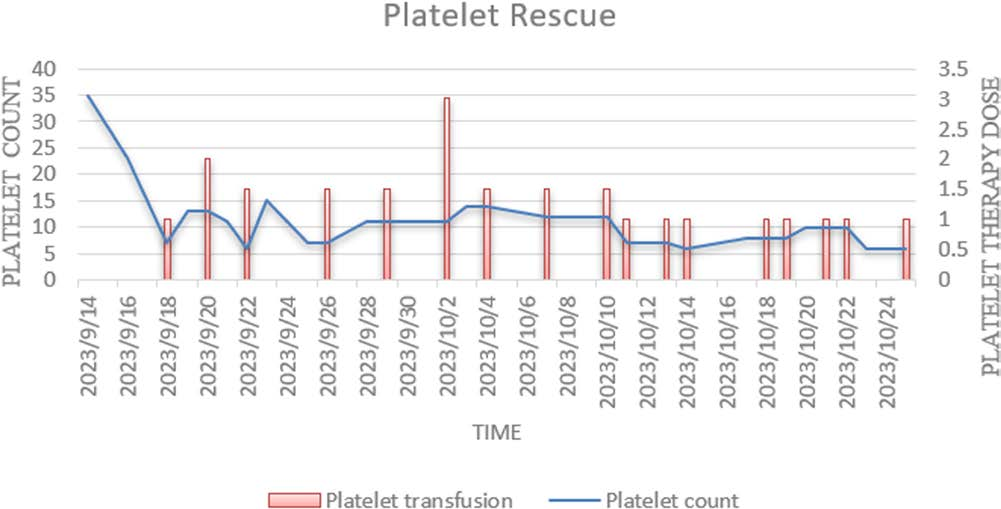
Longitudinal changes in platelet counts and corresponding volumes of platelet transfusions administered over time.

**Figure 4.**
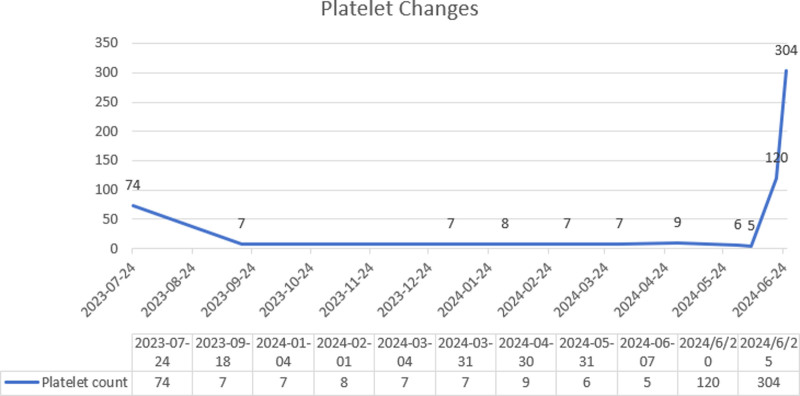
Platelet count trends following romiplostim N01 administration (250 µg subcutaneous injections on June 5, 2024 and June 16, 2024), demonstrating rapid recovery and sustained levels.

Subsequent treatment with fulvestrant monotherapy for 2 months resulted in a significant reduction in liver lesions (partial response) but failed to improve platelet counts. A repeat bone marrow biopsy showed persistent absence of megakaryocytes. The patient was briefly treated with abemaciclib, but this was discontinued due to severe CTIT, and she resumed fulvestrant monotherapy. By May 2024, platelet counts remained critically low at 5–10 × 10^9^/L, with no appreciable response to prior thrombopoietic therapies (Figs. 3 and 4). Besides, disease progression was observed, with significant enlargement of liver lesions compared to prior imaging. Chemotherapy was deemed unsuitable due to impaired bone marrow function, and treatment was switched to letrozole.

**Figure 5.**
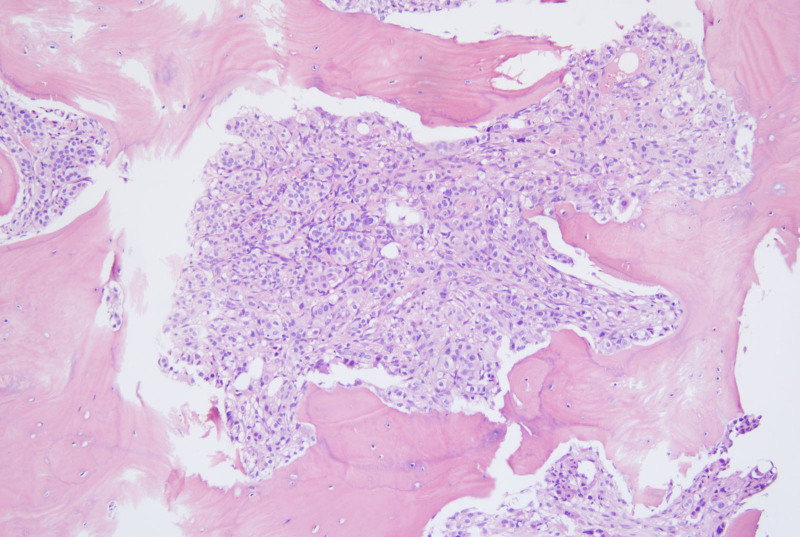
Bone marrow biopsy from the right iliac crest (July 2024), revealing fibroconnective tissue and atypical glandular hyperplasia arranged in glandular tubes, consistent with metastatic disease (magnification ×100).

In June 2024, the patient’s platelet count had fallen to 5 × 10^9^/L. On June 5, 2024, romiplostim N01 (250 µg, subcutaneous) was initiated for CTIT management, followed by a second dose on June 16, 2024. Platelet counts improved markedly, rising to 120 × 10^9^/L by June 20, 2024, with sustained levels between 251 × 10^9^/L and 304 × 10^9^/L thereafter (Fig. 4). A right iliac crest bone marrow biopsy in July 2024 revealed fibroconnective tissue and atypical glandular hyperplasia arranged in glandular tubes, consistent with metastatic moderately differentiated adenocarcinoma (Fig. 5); reticulin staining was strongly positive (3+). Platelet morphology appeared normal, with evidence of clustered distribution.

With platelet counts restored to normal levels and HER2 expression confirmed as low (1+), the patient was considered at high risk for combination chemotherapy toxicity. After discussion with the patient and family, treatment with the antibody-drug conjugate DS-8201 was initiated on July 7, 2024. The therapy was well-tolerated, and by August 2024, the tumor status was reassessed as stable disease, with the patient in good general condition. Regular follow-up revealed no recurrence of severe thrombocytopenia, and the patient continued treatment uneventfully.

The study adheres to the Declaration of Helsinki, written consent to publish this article has been obtained from the patient.

## 3. Discussion

CTIT imposes a significant burden on cancer care by frequently necessitating dose reductions, treatment delays, or chemotherapy discontinuation, all of which compromise relative dose intensity.^[3]^ Reduced dose intensity has been consistently linked to poor outcomes, especially in advanced malignancies.^[5,6]^ In China, therapeutic options for CTIT are limited to platelet transfusion and platelet-producing drugs such as rhTPO and rhIL-11.^[4]^ While platelet transfusions offer transient benefits and carry risks such as alloimmunization,^[7]^ rhTPO effectively stimulates platelet production but is mainly used in China and lacks global guideline endorsement. In our case, despite dose reductions and treatments including rhTPO, eltrombopag, avatrombopag, steroids, and platelet transfusions, her platelet count remained critically low. Romiplostim N01 was subsequently administered, resulting in rapid platelet recovery and sustained levels, allowing for uninterrupted cancer treatment.

TPO-RAs represent a promising class of drugs under investigation for CTIT. These agents activate the TPO receptor without incorporating the peptide sequence of endogenous TPO.^[8]^ Currently, 5 TPO-RAs are available: romiplostim, a peptibody administered subcutaneously,^[9]^ and the orally available small molecules eltrombopag,^[10]^ avatrombopag,^[11]^ lusutrombopag^[12]^ and hetrombopag.^[13]^ TPO-RAs are approved for indications such as immune thrombocytopenia, hepatitis C-associated thrombocytopenia, and aplastic anemia.^[14]^ Early studies of TPO-RAs in CTIT suggest varying degrees of efficacy, with romiplostim showing the most consistent results.^[15]^ Eltrombopag studies failed to demonstrate significant improvements in platelet nadirs compared to placebo, partly due to the confounding effect of spontaneous platelet recovery in control groups.^[16,17]^ Similarly, avatrombopag did not meet its composite primary endpoint in a phase 3 trial for CTIT prevention, likely reflecting the challenges of managing nadir CTIT, where transient platelet reductions often resolve spontaneously.^[18]^ Romiplostim, in contrast, has demonstrated encouraging efficacy in CTIT, particularly in cases of persistent thrombocytopenia. Multiple phase 2 studies have shown rapid and sustained platelet recovery with weekly romiplostim, enabling uninterrupted chemotherapy in most patients.^[19–21]^ In 1 randomized trial, 93% of romiplostim-treated patients achieved platelet counts ≥ 100 × 10^9^/L within 3 weeks compared to 12.5% in the control group.^[19]^ Additionally, retrospective analyses have reported reduced chemotherapy delays, fewer platelet transfusions, and maintenance of dose intensity in romiplostim-treated patients.^[22]^ Although no TPO-RAs have received regulatory approval for CTIT. Nevertheless, romiplostim is recommended in the National Comprehensive Cancer Network guidelines as an option for CIT,^[23]^ reflecting its potential utility in this challenging clinical scenario.

The limited efficacy of conventional thrombopoietic agents in this case may be attributed to multiple factors. Bone marrow biopsies consistently demonstrated a profound absence of megakaryocytes alongside infiltration by metastatic adenocarcinoma, suggesting suppression of the hematopoietic niche. Moreover, grade 3 reticulin fibrosis highlighted a fibrotic microenvironment known to impair megakaryopoiesis and hematopoietic stem cell activity. Prior exposure to myelotoxic regimens, including AC-T and docetaxel-capecitabine, likely resulted in cumulative marrow damage and stem cell depletion. Agents such as eltrombopag and avatrombopag rely on residual megakaryocyte precursors for efficacy and, thus, are ineffective when the marrow is functionally exhausted. Unlike eltrombopag, avatrombopag, or endogenous thrombopoietin, romiplostim activates the TPO receptor through a distinct binding domain that does not mimic the native ligand. This property mitigates the risk of competitive inhibition from circulating endogenous TPO.^[9]^ Moreover, romiplostim’s activation of downstream JAK-STAT and MAPK pathways is robust and sustained, promoting both megakaryocyte proliferation and platelet production.^[24]^ Unlike eltrombopag, romiplostim is not metabolized by the liver, an important consideration in cancer patients with hepatic dysfunction or liver metastases, which may explain its superior efficacy in this case.^[8]^ Eltrombopag, an oral TPO-RA, requires strict dietary restrictions and is associated with variable bioavailability due to interactions with polyvalent cations in food or supplements.^[10]^ Similarly, avatrombopag, while offering more consistent pharmacokinetics, has shown limited benefit in clinical trials for CTIT, particularly in cases of persistent thrombocytopenia where endogenous bone marrow function is severely impaired.^[18]^ In contrast, romiplostim’s subcutaneous administration ensures predictable absorption, and its extended half-life supports sustained TPO receptor engagement, making it a more reliable option for refractory CTIT.^[9]^

This case highlights the effectiveness of romiplostim N01 in treating refractory CTIT, enabling the continuation of cancer therapy without delays or dose reductions. Persistent CTIT presents a significant challenge to treatment progression and patient outcomes, particularly when conventional approaches fail. Romiplostim N01’s distinct mechanism of action allows for rapid platelet recovery, bypassing limitations of other thrombopoietic agents, and reducing the need for platelet transfusions. Its favorable safety profile and effectiveness suggest that romiplostim N01 can serve as a valuable alternative for managing refractory CTIT, improving patient quality of life and supporting uninterrupted cancer therapy.

## Author contributions

**Writing – original draft:** Peng Liu, Bo Xie, Jiajun Ling.

**Data curation:** Enlai Dong, Yiming Jiang, Lin Chen.

**Formal analysis:** Enlai Dong, Yiming Jiang.

**Writing – review & editing:** Bo Xie, Jiajun Ling.
